# The *FCRL3* −169T>C polymorphism and the risk of endometriosis-related infertility in a Polish population

**DOI:** 10.1007/s00404-013-2829-5

**Published:** 2013-04-04

**Authors:** Malgorzata Szczepańska, Przemyslaw Wirstlein, Hanna Hołysz, Jana Skrzypczak, Paweł P. Jagodziński

**Affiliations:** 1 Division of Reproduction, Department of Obstetrics, Gynecology and Gynecological Oncology, Poznań University of Medical Sciences, Poznań, Poland; 2 Department of Biochemistry and Molecular Biology, Poznań University of Medical Sciences, 6 Święcickiego St., 60-781 Poznań, Poland; 3 Department of Clinical Chemistry and Molecular Diagnostics, Poznań University of Medical Sciences, 49 Przybyszewskiego St., 60-355 Poznań, Poland

**Keywords:** Polymorphisms, Endometriosis, Infertility

## Abstract

**Objective:**

Recently, the *FCRL3* −169T>C (rs7528684) single-nucleotide polymorphism (SNP) has been demonstrated to be a risk factor of endometriosis related infertility. We studied whether the *FCRL* −169T>C SNP can be associated with endometriosis-related infertility in a sample of the Polish population

**Methods:**

Using PCR–RFLP analysis we genotyped 141 infertile women with endometriosis and 519 fertile women. FCRL3 transcript levels were determined by reverse transcription and real-time quantitative PCR analysis in CD19^+^ B cells from women with endometriosis-associated infertility and fertile women

**Results:**

We found a significantly increased frequency of the *FCRL3* C/C genotype in women with endometriosis-associated infertility than controls [OR = 1.681 (95 % CI = 1.120–2.522, *p* = 0.0116, *p*_corr_ = 0.0348)]. There was also a statistically increased frequency of the C/C and C/T genotypes in patients compared with controls [OR = 2.009 (95 % CI = 1.214–3.324, *p* = 0.0059, *p*_corr_ = 0.0177)]. The *p* value of the *χ* ^2^ test for the trend observed for the *FCRL3* −169T>C polymorphism was also statistically significant (*p*_trend_ = 0.0012, *p*_corr_ = 0.0036). We also found significantly increased FCRL3 transcript levels in carriers of the *FCRL3* −169 CC vs TT and CT vs TT genotype both in women with endometriosis-related infertility (*p* = 0.012; *p* = 0.015) and fertile women (*p* = 0.017; *p* = 0.032)

**Conclusions:**

*FCRL3* −169T>C polymorphism alters the expression of *FCRL3* and can be a risk factor of endometriosis-related infertility.

## Introduction

Endometriosis is a gynecological disorder that develops in approximately 6–10 % of reproductive age women [1]. This disease is attributed to the presence of ectopic endometrial tissue in the abdominal organs and the abdominal cavity [1, 2]. Although endometriosis has been intensively studied, the exact cause of this disorder and associated infertility is still unclear [1–3]. Involvement of the genetic background, environmental factors, and changes in the endocrine and immune systems are recognized in the development and persistence of endometriosis [1–3].

The growth of endometrium that implants outside the uterine cavity in women with endometriosis can be explained by two main findings. First, the endometriotic implants display activation of oncogenic pathways or increased activity of estrogen action, as well as prostaglandins, cytokines, and metalloproteinases supporting their survival [4]. Second, the ability of endometrium implants to survive outside the uterus may be linked to a defective immune system that is unable to clear these endometrial implants [4, 5].

It has been demonstrated that reduced natural killer cell cytotoxicity and cytokines biosynthesized by peritoneal macrophages may increase implantation and growth of ectopic endometrial tissue [6–8]. There is also evidence that B lymphocytes play a significant role in the pathogenesis of endometriosis [9]. They produce autoantibodies, which are commonly seen in various autoimmune diseases [9]. This indicates that genetic factors that modulate the function of B cells may be associated with endometriosis and endometriosis-related infertility.

Recently, the *FCRL3* gene, encoding Fc receptor-like 3 (*FCRL3*), was suggested as a novel autoimmune risk factor [10]. FCRL3 is an orphan cell surface receptor presented mainly in B lymphocytes of lymph nodes and germinal centers [11]. The single-nucleotide polymorphism (SNP) −169T>C (rs7528684), situated in the promoter region, changes the expression of *FCRL3* [10]. In addition to this finding, Kochi et al. (2008) demonstrated the *FCRL3* 169C gene variant effect on the inhibitory potential of B cell receptor-mediated signaling that alters the activation threshold and promotes tolerance breakdown in B cells [11]. Recently, the *FCRL3* −169T>C SNP was demonstrated as a risk factor of endometriosis related infertility [12]. Therefore, we studied whether the *FCRL3* −169T>C SNP may be associated with endometriosis-related infertility in a sample of the Polish population.

## Materials and methods

### Patients and controls

Peripheral blood samples from women with endometriosis and control women were obtained from the Gynecologic and Obstetrical University Hospital, Division of Reproduction at Poznan University of Medical Sciences. The studied women were allocated into one of two groups: 141 were primary infertility women with endometriosis and 519 women were used as the fertile controls (Table 1). Inclusion criteria for infertile women with endometriosis were regular menses, no anatomical changes in the reproductive tract, no hormonal treatments, and a minimum of 1 year of infertility with a current desire for conception. Exclusion criteria were male factor infertility, polycystic ovary syndrome (PCOS), mechanical distortion of the endometrial cavity by fibroids, and bilateral tubal occlusion. All included patients with endometriosis had laparoscopic and histological diagnosis of endometriosis. The stage of endometriosis was assessed according to the revised classification of the American Society for Reproductive Medicine (rASPM) [13].

**Table 1:** Clinical characteristics of women with endometriosis and controls

Characteristic	Endometriosis	Controls
Numbers	141	519
Age (years)	33 (21–38)^a^	33 (20–39)^a^
Parity	NA	1 (1–4)^a^
Duration of infertility (years)	4 (1–8)^a^	NA
RASRM (stage)^b^	Stage I (*n* = 69)	NA
	Stage II (*n* = 72)	

*NA* not applicable

Median ^a^(Range), revised American Society for Reproductive Medicine classification ^b^(rASRM) [13]

The fertile women assigned to the control group were examined for the cause of chronic pelvic pain without any pelvic abnormalities determined by laparoscopy. The controls were diagnosed as having varicose veins in the pelvic floor but no signs of past or present inflammation. Inclusion criteria for fertile control women were regular menses, no anatomical changes in the reproductive tract, no hormonal treatments, and at least one child born no more than 1 year before laparoscopy (Table 1). Exclusion criteria were diagnosis of past or present inflammation, pelvic abnormalities, endometriotic lesions, PCOS, and bilateral tubal occlusion. All included control women were laparoscopically examined due to chronic pelvic pain and suspected endometriosis, pelvic floor varicose veins. Patients and controls were matched by age and were all Caucasian of Polish descent (Table 1). Written informed consent was obtained from all participating individuals. The study procedures were approved by the Local Ethical Committee of Poznan University of Medical Sciences.

### Genotyping

DNA was isolated from peripheral leucocytes using a standard salting out procedure. Identification of the *FCRL3* −169T>C (rs7528684) polymorphic variant was performed by polymerase chain reaction-restriction fragment length polymorphism (PCR–RFLP). PCR was conducted employing primer pair 5′CTGAACAGGAAAATAATACAAATGT 3′and 5′TGAAACAAAATAATGGGGTGGAA 3′. The PCR-amplified fragments of *FCRL3* that were 167 bp in length were isolated and digested with the endonuclease BsmFI (5′ GGGAC(N)_10_/3′) New England BioLabs (Ipswich, USA). The *FCRL3* C allele was cleaved into 119 and 48 bp fragments, whereas the *FCRL3* T allele remained uncut. DNA fragments were separated by electrophoresis on 3 % agarose gel and visualized by ethidium bromide staining. The *FCRL3* −169T>C polymorphism was confirmed by repeated PCR–RFLP for all samples. Furthermore, 20 % of randomly selected samples were confirmed by commercial sequencing analysis.

### CD19^+^ B cell isolation

A 5-ml blood sample from women with endometriosis related infertility or controls was collected into tubes containing EDTA. To obtain CD19^+^ B cells from whole peripheral blood, we used the positive biomagnetic separation technique using Dynabeads^®^ CD19 Pan B, Dynal Biotech (Oslo, Norway) according to the manufacturer’s instructions. Magnetic beads were detached by DETACHaBEAD^®^ CD19, Dynal Biotech (Oslo, Norway) using a polyclonal sheep anti-mouse-Fab antibody. Isolated CD19^+^ cells were washed twice at room temperature in modified Eagle’s medium, followed by total RNA isolation.

### Real-time quantitative PCR (RQ-PCR) analysis of FCRL3 transcript level in CD19^+^ B cells

Total RNA was isolated according to the method of Chomczyński and Sacchi [14]. RNAs samples were treated with DNase I, quantified and reverse-transcribed (RT) into cDNA. Quantitative analysis of FCRL3 cDNA was performed by Light Cycler^®^480 II Real-Time PCR System, Roche Diagnostics GmbH (Mannheim, Germany), using SYBR Green I as detection dye. FCRL3 cDNA was quantified using the relative quantification method with a calibrator. The calibrator was prepared as a cDNA mix from all cDNA samples and consecutive dilutions were used to create a standard curve as described in Relative Quantification Manual Roche Diagnostics GmbH (Mannheim, Germany). For amplification, 1 μl of cDNA solution was added to 9 μl of LightCycler 480 SYBR Green I Master Mix Roche Diagnostics GmbH (Mannheim, Germany) and primers. The quantity of FCRL3 transcript in each sample was standardized by the porphobilinogen deaminase (PBGD) transcript level. The PCR amplification efficiency for target and reference genes was determined by different standard curves created by consecutive dilutions of the cDNA template mixture, as provided in Relative Quantification Manual Roche Diagnostics GmbH (Mannheim, Germany). The FCRL3 cDNA 123 bp amplicon was amplified employing the primer pair: (forward 5′ GGTCACAGTTCCGGTGTCTC3′) and (reverse 5′ CAGTACAGGATCGGGAAGGA3′). The PBGD cDNA 160 bp amplicon was amplified using the primer pair: (forward 5′ GCC AAG GAC CAG GAC ATC 3′) (reverse 5′ TCA GGT ACA GTT GCC CAT C 3′). The FCRL3 mRNA levels were expressed as multiples of these cDNA concentrations in the calibrator.

### Statistical analysis

The distribution of genotypes in patients and controls was examined for deviation from Hardy–Weinberg equilibrium. Since the best inheritance model contribution of *FCRL3* −169T>C SNP to endometriosis-related infertility is unknown in our population, the data were analyzed under recessive and dominant models. In addition, to determine the polymorphism’s association with endometriosis-related infertility, the additive genetic model was tested using Armitage’s test for trend (*p*_trend_). The differences in genotype frequencies between cases and controls were determined using standard Chi squared (*χ* ^2^), the Odds ratio (OR), and 95 % Confidence intervals (95 % CI) were calculated and a *p* value <0.05 was considered statistically significant. Power calculations for this study were conducted using Quanto software (Gauderman WJ, Morrison JM. QUANTO 1.2: A computer program for power and sample size calculations for genetic-epidemiology studies, URL: http://hydra.usc.edu/gxe, 2006). Statistical analysis of FCRL3 transcript level comparison between CC vs TT and CT vs TT genotype carriers was evaluated by Mann–Whitney Rank Sum Test.

## Results

### Distribution of the *FCRL3* −169T>C polymorphism in women with endometriosis associated infertility

Genotype evaluation of the *FCRL3* −169T>C polymorphism did not demonstrate significant aberration from the Hardy–Weinberg equilibrium in the patient and control groups.

We found an approximately 1.4-fold significantly increased frequency of the *FCRL3* C/C genotype in in women with infertility-associated endometriosis over the controls [OR = 1.681 (95 % CI = 1.120–2.522, *p* = 0.0116, *p*_corr_ = 0.0348)]. There was also a significantly increased frequency of the C/C and C/T genotypes in patients compared with controls [OR = 2.009 (95 % CI = 1.214–3.324, *p* = 0.0059, *p*_corr_ = 0.0177)] (Table 2). The *p* value of the *χ* ^2^ test for the trend observed for the *FCRL3* −169T>C polymorphism was also statistically significant (*p*_trend_ = 0.0012, *p*_corr_ = 0.0036).

**Table 2:** Association of the *FCRL3* −169T>C (rs7528684) SNP with endometriosis-related infertility

Gene (rs no.)	Alleles^a^	MAF^b^	Genotypes^c^			OR_recessive_ (95 % CI); *p* value^d^	OR_dominant_ (95 % CI); *p* value^d^	*p*_trend_ value^e^
			Cases		Controls			
*FCRL3* (rs7528684)	c/T	0.49	no	21/73/47	135/265/119	1.681 (1.120–2.522); 0.0116	2.009 (1.214–3.324); 0.0059	0.0012
			%	15,52,33	26,51,23			

^a^Uppercase denotes the more frequent allele in the control samples

^b^MAF, minor allele frequency calculated from the control samples

^c^The order of genotypes: DD/Dd/dd (d is the minor allele)

^d^Chi square analysis

^e^Cochran-Armitage trend test

A power analysis predicted sufficient power to detect an association of the *FCRL3* −169T>C polymorphism with a genetic effect of 2.00 or more for the dominative model and a genetic effect of 1.80 or more for the recessive model (Table 3).

**Table 3:** Power analysis

	Model	
Genetic effect	Dominant	Recessive
1.00	0.0500	0.0500
1.20	0.1300	0.1342
1.40	0.3153	0.3532
1.60	0.5239	0.6119
1.80	0.6967	0.8106
2.00	0.8169	0.9224
2.20	0.8925	0.9723

Power calculations for this study were conducted using Quanto software (Gauderman WJ, Morrison JM. QUANTO 1.2: A computer program for power and sample size calculations for genetic-epidemiology studies, URL: http://hydra.usc.edu/gxe, 2006). Power analysis was conducted with the following parameters: allele frequency 0.49, frequency of endometriosis associated infertility 0.03 in population, significance level 0.05, two-sided

### Effect of *FCRL3* −169T>C polymorphism on FCRL3 transcript levels in CD19^+^ B cells from fertile women and women with endometriosis-related infertility

**Fig. 1:**
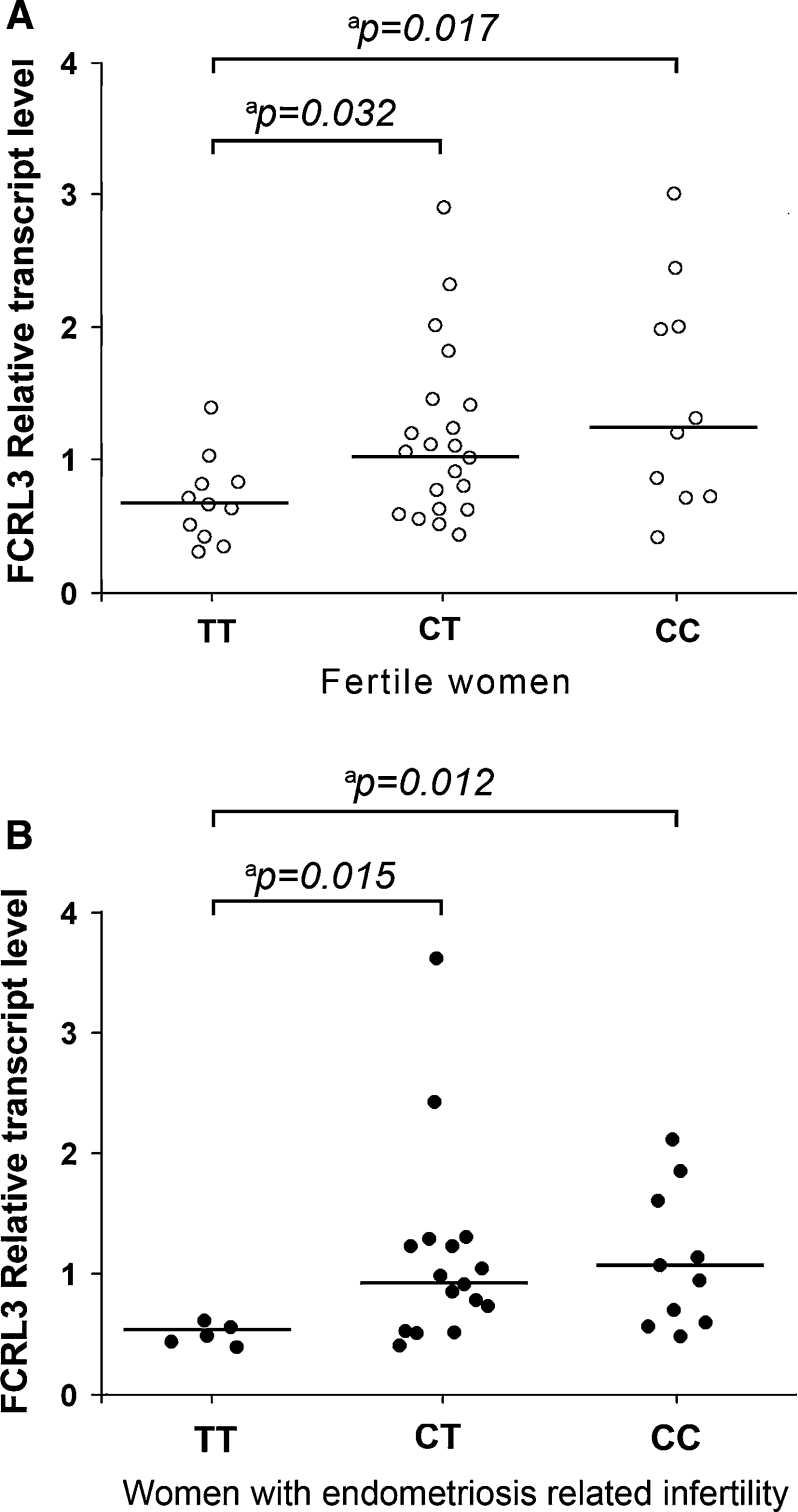
Effect of the *FCRL3* −169T>C polymorphism on FCRL3 transcript levels in CD19^+^ B cells in fertile women (**a**) and in women with endometriosis related infertility (**b**). The CD19^+^ B cells from whole venous peripheral blood were isolated using positive biomagnetic separation technique. Quantitative analyses of FCRL3 transcript levels were performed by RT and RQ-PCR SYBR Green I system. The *open circles* correspond to transcript levels in fertile women, carriers of CC, CT, and TT genotypes. The *black circles* represent FCRL3 transcript levels in CD19^+^ B cells from women with endometriosis-associated infertility, carriers of CC, CT, and TT genotypes. ^a^Mann-Whitney Rank Sum Test

We observed significantly increased FCRL3 transcript levels in the CD19^+^ B cells from carriers of the *FCRL3* −169 C gene variant as compared with carriers of the *FCRL3* −169 TT genotype in fertile women. The median of FCRL3 transcript levels in B cells from fertile women with the CC genotype was 1.263 (range 0.305–0.421) (*p* = 0.017), 1.060 (range 0.430–2.899) (*p* = 0.032) for the CT genotype, and 0.663 (range 0.301–1.349) for the TT genotype (Fig. 1a). There were also significantly increased FCRL3 transcript levels in the CD19^+^ B cells from carriers of the *FCRL3* −169 C gene variant as compared with carriers of the *FCRL3* −169 TT genotype in women with endometriosis-related infertility. The median of FCRL3 transcript levels in B cells from endometriosis women with the CC genotype was 1.017 (range 0.493–2.120) (*p* = 0.012), with the CT genotype was 0.951 (range 0.406–2.616) (*p* = 0.015), and with the TT genotype was 0.485 (range 0.391–0.611) (Fig. 1b).

## Discussion

Endometriosis is a complex disease accompanied by changes in the expression of genes encoding proteins affecting sex hormone activity, proteins involved in vascular and tissue rebuilding, glucose homeostasis enzymes, and inflammatory mediators [15]. Infertility is recognized in 30 to 50 % of women with endometriosis [1–3]. This disorder disturbs most stages of reproduction, including granulosa cell steroidogenesis, folliculogenesis, ovulation, fertilization, and blastocyst implantation [1, 16, 17].

There are several pieces of evidence that demonstrate the involvement of an abnormal humoral immune response in the infertility of women with endometriosis. In patients with endometriosis, B cells have been shown to produce autoantibodies recognizing phospholipids, histones, polynucleotides, and even autoantibodies characteristic for lupus [18, 19]. The presence in blood plasma of autoantibodies against endometrial, ovarian, and laminin-1 tissues may partially explain subfertility in women with endometriosis [20, 21]. Moreover, women with endometriosis exhibit headaches, arthralgia, myalgia, fibromyalgia, chronic fatigue syndrome, and other symptoms characteristic for patients with autoimmune disease [22–24]. This suggests common candidate genes that contribute to the pathogenesis of autoimmunity and endometriosis [25].

We observed an association of the *FCRL3* −169T>C polymorphism with occurrence of endometriosis related infertility. To date, the rs7528684 SNP has been recognized as a genetic risk factor of endometriosis-related infertility as well as idiopathic infertility in Brazilian women [12, 26, 27].

The *FCRL3* −169T>C polymorphism has also been identified as a risk factor of various autoimmune diseases, including rheumatoid arthritis, systemic lupus erythematosus, Hashimoto thyroiditis, Graves’ disease, and biliary cirrhosis [10, 28].

We also found significantly increased FCRL3 transcript levels in the CD19^+^ B cells from carriers of the *FCRL3* −169T C gene variant as compared with carriers of the *FCRL3* −169 TT genotype both in women with endometriosis-related infertility and fertile women. To date, it has been observed that the *FCRL3* −169T>C SNP affects the expression of *FCRL3* by changing the affinity binding of nuclear factor-kappa B (NF-κB) to its promoter. Kochi et al. [10], using luciferase reporter gene analysis, demonstrated the significantly increased transcriptional activity of the disease-susceptible *FCRL3* −169C gene variant as compared with the non-disease-susceptible *FCRL3* −169T gene variant. Moreover, Gibson et al. [29] demonstrated that venous blood CD19^+^ B cells from normal donors, −169 CC homozygotes, exhibited significantly higher levels of the FCRL3 protein than B cells from TT homozygotes.

To date, the genetic risk factors for endometriosis-related infertility have included the *17β*-*hydroxysteroid dehydrogenase type 1*, *ESR1*, *ESR2*, and *luteinizing hormone beta*-*subunit* genes [30–33]. Furthermore, polymorphisms situated in *FOXP*, *complement component 3,* and *lysyl oxidase*-*like protein 4* genes have also been reported as genetic factors which affect fertility in women with endometriosis [27, 34].

Our study confirmed an association of the *FCRL3* −169T>C polymorphism with endometriosis-related infertility. Our genetic studies were conducted on a limited number of infertile women with endometriosis; therefore, the role of this polymorphism should be further studied in women with idiopathic infertility and a larger and independent cohort of women with endometriosis-related infertility.
